# Sphingosine-1-Phosphate and Its Effect on Glucose Deprivation/Glucose Reload Stress: From Gene Expression to Neuronal Survival

**DOI:** 10.1007/s12035-014-8807-5

**Published:** 2014-07-24

**Authors:** Kinga Czubowicz, Magdalena Cieślik, Joanna Pyszko, Joanna B. Strosznajder, Robert P. Strosznajder

**Affiliations:** 1 Laboratory of Preclinical Research and Environmental Agents, Department of Neurosurgery, Mossakowski Medical Research Centre, Polish Academy of Sciences, 5 Pawińskiego Street, 02-106 Warsaw, Poland; 2 Department of Cellular Signaling, Mossakowski Medical Research Centre, Polish Academy of Sciences, 5 Pawińskiego Street, 02-106 Warsaw, Poland

**Keywords:** Gucose deprivation, Sphingolipids, HT22, S1P, Sphingosine kinase 1, Bcl-2 proteins

## Abstract

Sphingosine kinase-1 (Sphk1-1, EC 2.7.1.91) is a regulator of pro-survival signalling, and its alterations have been observed in Alzheimer’s disease, brain ischemia and other neurological disorders. In this study we addressed the question whether Sphk1 and its product, sphingosine-1-phosphate (S1P), play a significant role in glucose deprivation (GD)/glucose reload (GR) stress in hippocampal neuronal cells (HT22). It was found that GD (6 h) followed by 24 h of GR evoked enhancement of the free radical level and neuronal HT22 cell death. Moreover, the significantly stronger gene expression for the pro-apoptotic Bax protein and down-regulation of the anti-apoptotic Bcl-2 and Bcl-X_L_ proteins were observed. Concomitantly, this stress up-regulated: gene expression, protein level and activity of Sphk1. Exogenous S1P at 1 μM concentration and the other agonists of the S1P1 receptor (SEW 2871 and P-FTY720) enhanced HT22 cell viability affected by GD/GR stress. This mechanism is mediated by S1P receptor(s) signalling and by the activation of gene expression for Bcl-2 and Bcl-X_L_. Summarising, our data suggest that sphingolipid metabolism may play an important role in the early events that take place in neuronal cell survival/death under GD/GR stress. Our data demonstrate that exogenous S1P, through the activation of specific receptors S1P1 and S1P3 signalling pathways, regulates the gene expression for anti-apoptotic proteins and enhances neuronal cell survival affected by GD/GR stress.

## Introduction

Homeostasis of blood and cellular glucose is very important for the functioning of the central nervous system (CNS). Moderate hypoglycemia impairs neuronal functions, but severe hypoglycemia causes the death of vulnerable cells [1–3]. Despite intense efforts in research, the mechanism of neuronal cell death evoked by glucose deprivation (GD) followed by glucose reload (GR) is not yet fully understood. Previous data indicated the important role of excitotoxicity, but several other molecular events have also been suggested. It was observed that hypoglycemia induces a several-fold increase in glutamate and aspartate concentrations in the brain extracellular space [4]. Adenosine, which may reduce neuronal activity, can also increase during hypoglycemia [5, 6]. In addition, a Ca^2+^ and Zn^2+^ influx, production of reactive oxygen (ROS) and nitrogen (RNS) species and mitochondrial failure have been described to be associated with hypoglycemic neuronal damage [7, 8].

Studies on the regulation of cell survival and death presented recently focused on the important role of bioactive sphingolipids in these processes. Many data have shown the signalling functions of two sphingolipids, ceramide and sphingosine-1-phosphate (S1P). Numerous studies demonstrated that level of ceramide is enhanced by a variety of cell stressors. It was reported to increase along with brain aging, ischemia and in neurodegenerative disorders [9–12]. A high level of ceramides can be responsible for cell growth arrest and apoptosis [13, 14]. However, S1P may promote cell survival and proliferation [15, 16]. The balance between these two lipids (ceramide/S1P), which has been called the “sphingolipid-rheostat”, is controlled by sphingosine kinases (Sphk) [16, 17]. There are two isoforms of sphingosine kinases, Sphk1 and Sphk2, whose activities can be a crucial determinant of cell fate in stress responses.

S1P is implicated in both extracellular and intracellular-mediated signalling. S1P synthesized in the cell by sphingosine kinases is released, via the ABC family of transporters from the cell and is able to act in an autocrine or paracrine manner [18, 19]. The intracellular actions of S1P have been recently identified [20–24], for example nuclear S1P synthesized by sphingosine kinase 2 binds to and inhibits HDAC1 and HDAC2. Their inhibition by S1P prevents histone deacetylation and enhanced transcription of the genes encoding the cyclin-dependent kinase inhibitor p21 and the transcriptional regulator c-fos [20]. Also, intracellular S1P generated by sphingosine kinase 1 was shown to be necessary for TRAF2 E3 ubiquitin ligase activity, which is essential for TNF-mediated events [21].

The majority of S1P biological effects are known to be mediated by the activation of five specific G protein-coupled receptors (S1PR1–5) located on the cell surface. The S1P signalling through these receptors causes the activation of different downstream targets including: the Ras/ERK pathway to promote proliferation, the PI3K/Akt signalling to prevent apoptosis and the PI3K/Rac pathway involved with cytoskeletal rearrangement [24, 25].

Production of ROS can differently modulate Sphk1 and Sphk2 activity, depending on the severity of oxidative damage and the type of stress (ischemia, hypoxia and amyloid beta toxicity) [26–28]. Enhancement of Sphk1 activity at an early stage of stress signalling has a pro-survival effect by producing S1P and decreasing the ceramide concentration [29]. Sphk1/S1P can regulate cell viability and death by modulating the level of the Bcl-2 protein family [30, 31]. Until today, nothing is known about the role of Sphk1/S1P in GD/GR stress in hippocampal neuronal cells. The in vivo studies have indicated that hypoglycemic neuronal death is pronounced in hippocampal CA1, in the subiculum and dentate granule cell layer [32].

In our study we used mouse immortalised hippocampal neuronal cells (HT22) which had been already very well described [33] and widely applied in a large number of studies. The aim of this study was to examine the role of S1P in neuronal cell death evoked by GD/GR stress.

## Experimental Procedures

### Cell Culture

The studies were carried out using the mouse immortalised hippocampal nerve cell line HT22, which is a subclone of HT4. HT4 cells were immortalised from primary mouse hippocampal neurons using a temperature-sensitive small virus-40 T antigen [34, 35]. These cells retain the characteristics of differentiated neurons. HT22 cells were obtained from Professor David Schubert, Salk Institute, La Jolla, CA, USA. This cell line does not possess active ionotropic glutamate receptors and was very well described by Sagara and Schubert [33]. In accordance with this publication [33] HT22 cells express functional group I metabotropic glutamate receptors (mGluR1 and mGluR5). We recently used it extensively for different types of studies [36]. HT22 cells were cultured in 75-cm^2^ flasks in DMEM supplemented with 10 %-heat-inactivated fetal bovine serum (FBS), 1 % penicillin/streptomycin (50 U/ml) and 2 mM glutamine. Cells were maintained at 37 °C in a humidified incubator containing 5 % CO_2_. The HT22 cells were used for experiments between five and ten passage numbers. We kept HT22 cells at no greater than 50 % confluence. HT22 cells were seeded onto 96-well plate at a density of 2 × 10^3^ cells per well in 100 μl of medium. HT22 cells were seeded at 3 × 10^5^ cells/10-mm tissue culture dish.

### Glucose Deprivation/Glucose Reload

Equal cell numbers were seeded into 48-well or 96-well 0.1 % polyethyleneimine coated plates. After 24 h the cells were rinsed two times with phosphate-buffered saline (PBS) and treated with low-serum (2 % FBS) glucose-free medium (Gibco, Invitrogen). GD was terminated after 6 h by exchanging the glucose-free medium to low-serum (2 % FBS) Dulbecco’s modified Eagle medium (DMEM) supplemented with 25 mM glucose. The extent of cell death was assessed after a 24 h GR period by using MTT assay. S1P (1 μM) was added concomitantly with the initiation of GD. The cells were incubated with S1P (1 μM) under GD conditions and also during the recovery period. Osmolarity of the medium used in this study was measured by using the Osmomat 030 – Gonotec. Osmolarity determined in the glucose-free medium in either the absence and presence of 25 mM mannitol indicated small changes in osmolarity in the HT22 cells.

### Cell Viability Analysis

Mitochondrial function and cellular viability were evaluated using 2-(4,5-dimethylthiazol-2-yl)-2,5-diphenyltetrazolium bromide (MTT). After 6 h GD/24 hGR incubation with the appropriate compounds, MTT (2.5 mg/ml) was added to all of the wells. The cells were incubated at 37 °C for 2 h. Then the cells were lysed in DMSO and spectrophotometric measurement was performed at 595 nm.

### Determination of Free Radicals Using DCF Probe

ROS production in HT22 cells was assessed by using the 2′,7′-dichlorodihydrofluorescein diacetate (H2DCF-DA) probe [37, 38]. Cell media was changed after 6 h GD/24 h GR to Hanks’ buffer and incubation was continued in the presence of 10 μM H2DCF-DA for 50 min at 37 °C. Then fluorescence (excitation [*λ*_ex_], 485 nm; emission [*λ*_em_], 535 nm) was quantified.

### Determination of Gene Expression

Cells were washed twice with ice-cold PBS, scraped from the culture dish and centrifuged shortly (3 min, 1,000 × g). RNA was isolated from the cell pellet by using TRI reagent according to the manufacturer’s protocol (Sigma, St. Louis, MO, USA). Digestion of DNA contamination was performed by using DNase I according to the manufacturer’s protocol (Sigma). RNA quantity and quality were controlled by spectrophotometric analysis and gel electrophoresis. Reverse transcription was performed by using a High Capacity cDNA Reverse Transcription Kit according to the manufacturer’s protocol (Applied Biosystems, Foster City, CA, USA). Quantitative real time PCR was performed by using the following pre-developed TaqMan Gene Expression Assays (Applied Biosystems): Bcl-2-Mm00477631_m1, Bcl211-Mm00437783_m1, Bax-Mm00432051_m1, Sphk1-Mn00448841_g1, Actb-Mm00607939_s1 on an ABI PRISM 7500 apparatus according to the manufacturer’s instructions. Actin (Actb) was selected and used in all of the studies as a reference gene. The level of mRNA was calculated by ΔΔ*C*_t_ method.

### Immunochemical Determination of Protein Level (Western Blot)

After protein measurement according to Lowry, the homogenate of the HT22 cells was mixed with a 5 × Laemmli sample buffer and denatured for 5 min at 95 °C. Then, 40 μg of protein was loaded per lane on 10 % acrylamide gels and examined by SDS–PAGE. The proteins were transferred onto PVDF membranes at 100 V. Membranes were incubated in 5 % dry milk in TBS with Tween 20 (TBS-T) for 1 h and exposed overnight to the following antibodies: anti-Sphk1 (1:250, from Cell Signaling) and anti-GAPDH (1:4,000, from Sigma Aldrich). After treatment for 1 h with the corresponding horseradish peroxidase-coupled secondary antibodies (anti-rabbit from Sigma-Aldrich), the protein bands were detected by ECL reagent (ThermoScientific).

### Measurement of Sphk Activity

Sphk activity assay was performed according to a previous report [23]. After 24 h incubation, the cells were washed with iced PBS and lysed by a freeze–thaw cycle in 50 mM HEPES, pH 7.4, 10 mM KCl, 15 mM MgCl_2_, 0.1 % Triton X-100, 20 % glycerol, 2 mM orthovanadate, 2 mM dithiothreitol, 10 mM NaF,1 mM deoxypyridoxine, and EDTA-free complete protease inhibitor (Roche Applied Science). Lysates were cleared by centrifugation at 15,000 rpm for 5 min. The lysates and NBD-Sphingosine (10 μM final; Avanti Polar Lipids) were mixed in a reaction buffer (50 mM HEPES, pH 7.4, 15 mM MgCl_2_, 0.5 mM KCl, 10 % glycerol and 2 mM ATP) and incubated for 30 min at 30 °C. The reactions were stopped by the addition of an equal amount of 1 M potassium phosphate, pH 8.5, followed by an addition of 2.5-fold chloroform/methanol (2:1), and then centrifuged at 15,000 rpm for 1 min. Only the reactant NBD-S1P, but not the substrate NBD-Sphingosine, was collected in the alkaline aqueous phase. The fluorescence value was measured (*λ*_ex_ = 485 nm, *λ*_e_m = 538 nm) after the aqueous phase was combined with an equal amount of dimethylformamide [39].

### Determination of Apoptosis

Apoptosis was determined by Hoechst 33342 fluorescent staining. The cells were examined under a fluorescence microscope (Olympus BX51, Japan) and photographed with a digital camera (Olympus DP70, Japan). Cells with typical apoptotic nuclear morphology (nuclear shrinkage, condensation) were identified and counted. The results were expressed as percentages of apoptotic cells in the whole cell population.

### Statistical Analysis

The results were expressed as mean values ± SEM. Differences between means were analysed using Student’s *t*-test or one-way ANOVA followed by the Newman-Keuls test. Values of *p* < 0.05, *p* < 0.01 and *p* < 0.001 were considered statistically significant.

## Results

**Fig. 1:**
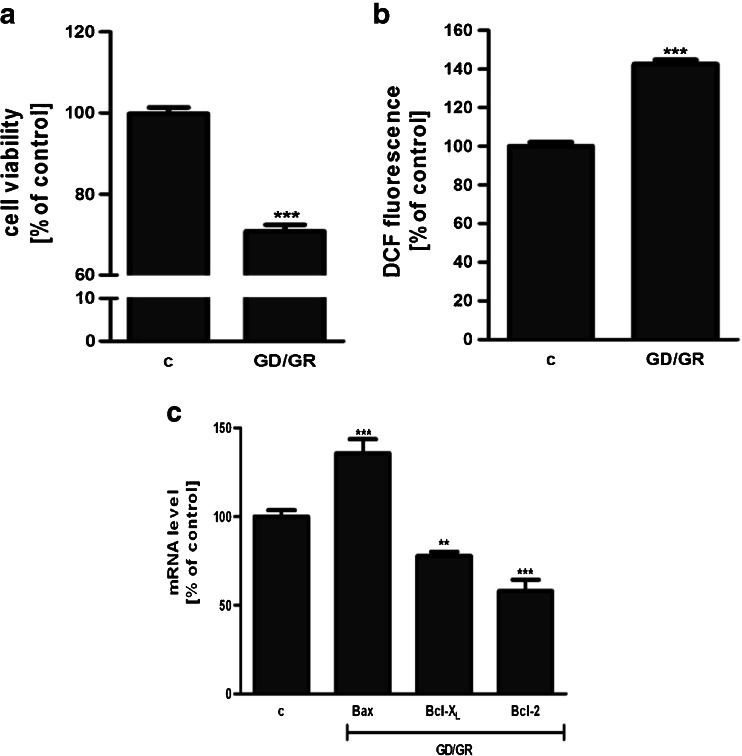
Effect of GD/GR on HT22 cell viability (**a**), ROS generation (**b**) and gene expression of pro- and anti-apoptotic Bcl-2 proteins (**c**). Data represent mean values ± SEM for four separate experiments with four to six replications (**a**) and for three separate experiments with three replications (**b**, **c**). ***p < 0.001 versus control [*c*] HT22 cells by Student’s *t*-test (**a**, **b**), ***p < 0.001, **p < 0.01 versus control [c] HT22 cells by one-way ANOVA followed by the Newman–Keuls test (**c**)

**Fig. 2:**
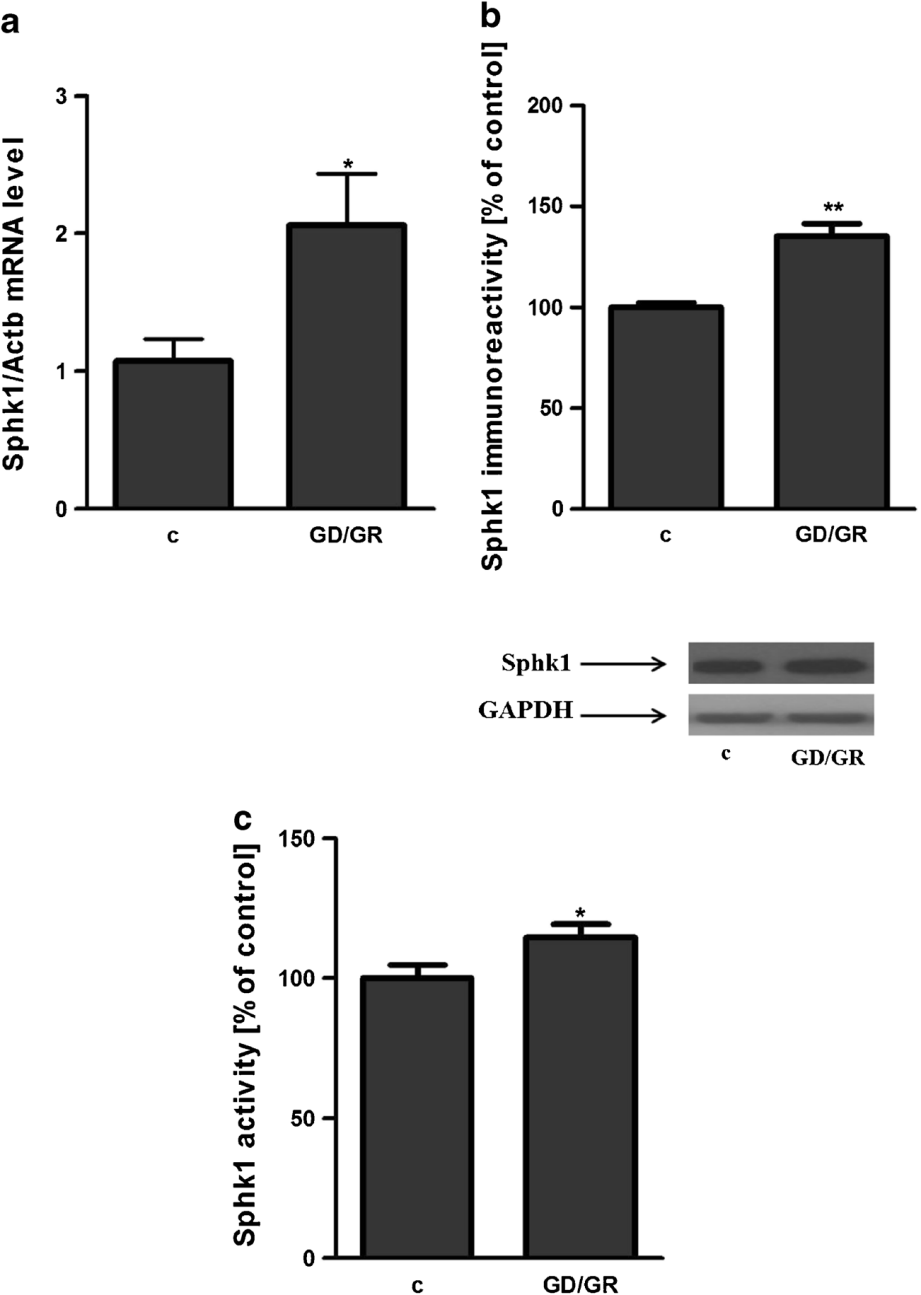
Sphingosine kinase activity, gene expression and protein level in hippocampal neuronal cells subjected to GD/GR stress. Data represent the mean value ± SEM for three separate experiments with three replications; *p < 0.05 versus control [*c*] HT22 cells by Student’s *t*-test (**a**, **c**). The absolute value of Sphk1 activity in control HT22 cells is 2655 [AU]. The absolute value of Sphk1 activity in GD/GR treated HT22 cells is 3044 [AU]. Data represent the mean value ± SEM for three separate experiments normalised against GAPDH, **p < 0.01 versus control [*c*] HT22 cells by Student’s *t*-test (**b**)

**Fig. 3:**
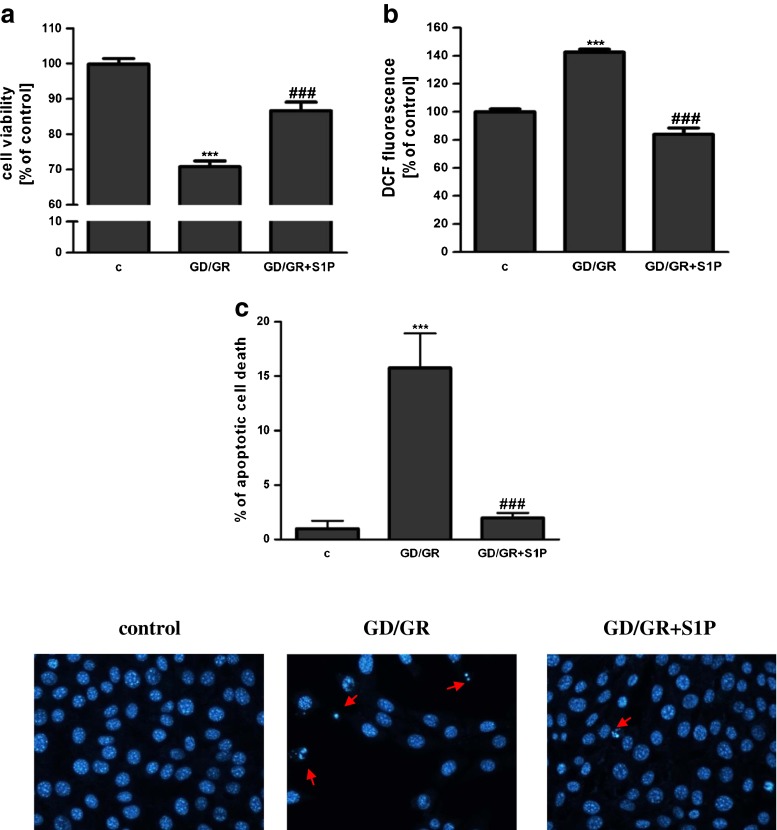
Effect of sphingosine-1-phosphate on HT22 cell viability (**a**) and ROS generation (**b**) after exposure to GD/GR. Data represent the mean value ± SEM for four separate experiments with four to six replications (**a**) and for three separate experiments with three replications (b). ***p < 0.001 versus control [c] HT22 cells, ^###^ *p* < 0.001 versus GD/GR-treated cells by one-way ANOVA followed by the Newman–Keuls test. Apoptosis induced by GD/GR and the protective effect of S1P in HT22 cells (**c**). The number of apoptotic cells was counted. Data represent the mean value ± SEM for three separate experiments with three replications, ***p < 0.001 versus control [*c*] HT22 cells, ^###^ *p* < 0.001 versus GD/GR-treated cells by one-way ANOVA followed by the Newman–Keuls test. The *arrows* indicate nuclei with typical apoptotic features

**Fig. 4:**
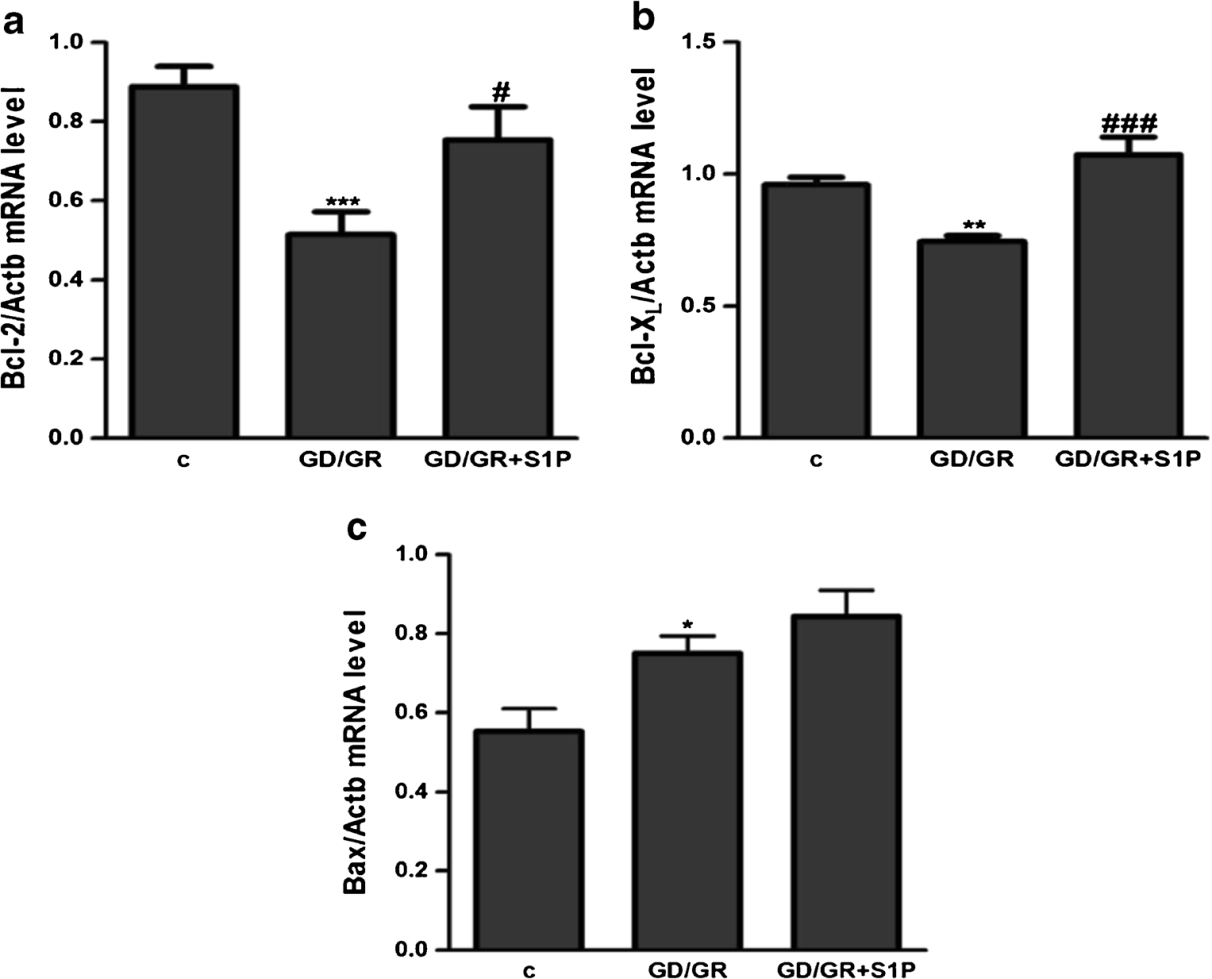
Effect of GD/GR and S1P on the expression of Bcl-2 (**a**), Bcl-X_L_ (**b**) and Bax (**c**) genes in HT22 cells. Data represent the mean value ± SEM for three separate experiments with three replications,*p < 0.05, **p < 0.01, ***p < 0.001 versus control [*c*] HT22 cells, ^#^ *p* < 0.05,  ^###^ *p* < 0.001 versus GD/GR-treated cells by one-way ANOVA followed by the Newman–Keuls test

**Fig. 5:**
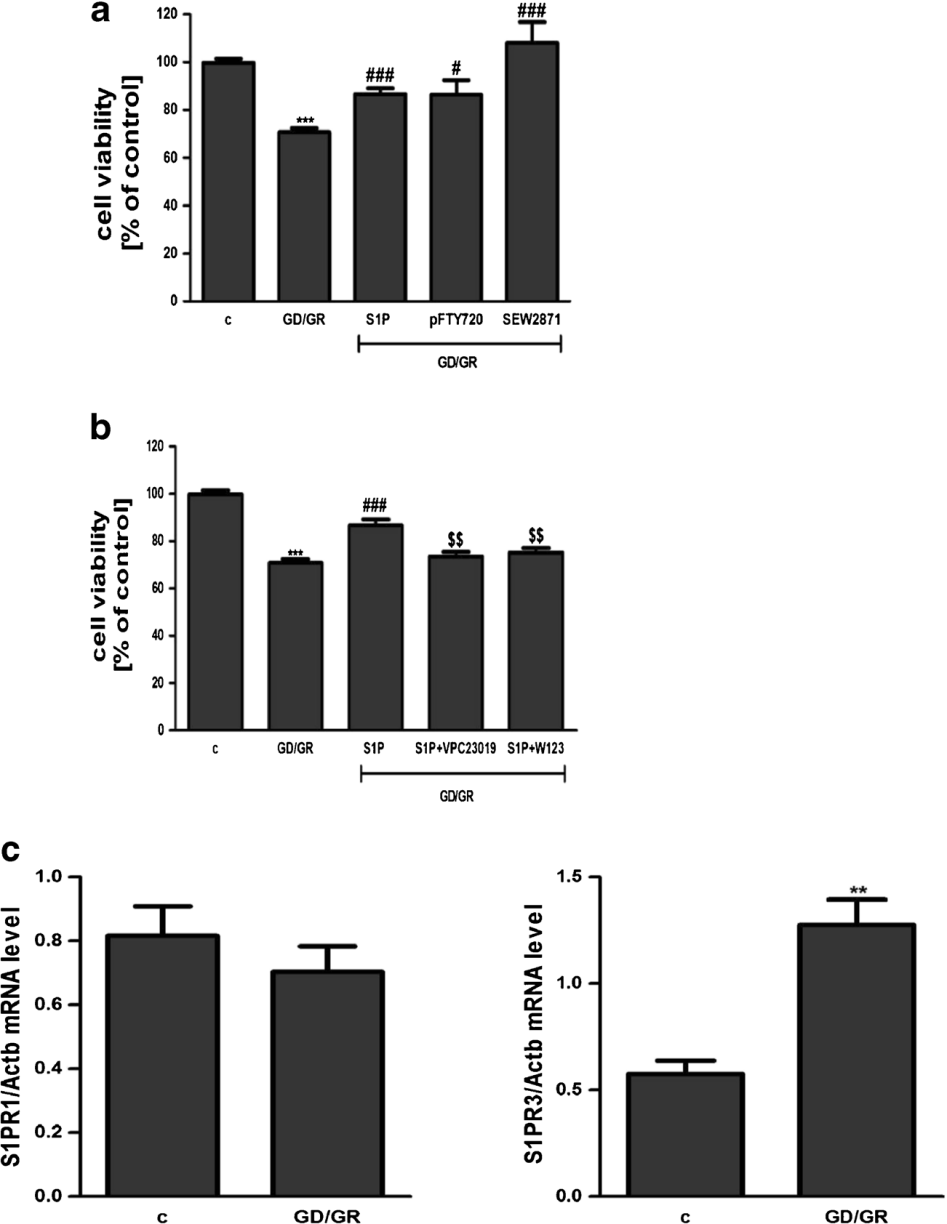
Effect of S1P analogue P-FTY720, S1P1 receptor agonist SEW2871 (**a**) and S1P receptor (1,3) antagonists (VPC23019 and W123) on HT22 cell viability (**b**), ***p < 0.001 versus control [*c*] HT22 cells, ^#^ *p* < 0.05, ^###^ *p* < 0.001 versus GD/GR-treated cells, ^$$^ *p* < 0.01 versus S1P-pretreated and GD/GR-treated cells by one-way ANOVA followed by the Newman–Keuls test. Effect of GD/GR and S1P on the expression of S1P1 and S1P3 (**c**). Data represent the mean value ± S.E.M for three separate experiments with three replications, **p < 0.001 versus control [*c*] HT22 cells by Student’s *t*-test

GD (6 h) followed by GR (24 h) decreased the viability of HT22 cells by about 30 %. The effect of osmotic changes on these cells’ survival was negligible. Our study presented that GD/GR leads to: significant cell death, enhancement of the free radical level and changes in the gene expressions of pro- and anti-apoptotic Bcl-2 proteins (Fig. 1a,b,c). Gene expression of the Hrk protein was observed at a very low level and was not altered by GD/GR (data not shown). GD/GR stress enhanced the gene expression, protein level and activity of Sphk1 (Fig. 2a,b,c). We also observed that exogenously added S1P significantly suppressed oxidative stress and increased HT22 cell survival affected by GD/GR (Fig. 3a,b). Microscopic examination of cell nuclei stained with DNA-binding fluorochrome Hoechst 33342 showed that HT22 cells exposed to GD/GR presented typical apoptotic morphology. The protective effect of S1P was seen in this fluorescence microscope study. This bioactive lipid significantly decreased death signalling evoked by GD/GR and the amount of apoptotic cells (Fig. 3c). Exogenous S1P at a concentration of 1 μM significantly enhanced the expression of Bcl-2 and Bcl-X_L_, both anti-apoptotic proteins suppressed by GD/GR. However, S1P did not alter GD/GR-evoked changes of Bax (Fig. 4a,b,c). Finally, we investigated whether the S1P effect is receptor-mediated. Using S1P receptor agonists, i.e., S1P, SEW 2871 (a specific agonist for S1P1) and P-FTY720 (an agonist for S1P1, S1P3, S1P4, S1P5), we demonstrated that the neuroprotective effect of S1P is mediated by activation of these receptors (Fig. 5a). These results were confirmed by using antagonists for S1P1 and S1P3 (VPC23019) and the specific antagonist for S1P1 (W123). Cell survival analysis indicated that both compounds abolished the protective effect of S1P on HT22 cells (Fig. 5b). Gene expression analysis demonstrated that the mRNA level of S1P3 under GD/GR stress was significantly increased and that the mRNA level of S1P1 was not changed (Fig. 5c). Based on these results we propose that the neuroprotective effect of S1P is mainly dependent on S1P1 and S1P3 receptor stimulation.

## Discussion

Although there are growing evidences that S1P receptor signalling may be neuroprotective in AD, brain injury, ischemia and other neurological disorders [40–44], there is limited data about its involvement in GD/GR stress. In this study, we used an in vitro model of metabolic-oxidative stress evoked by GD followed by GR, which is observed in the pathological conditions as mentioned above. GD/GR leads to a significant enhancement of free radical concentrations and HT22 hippocampal neuronal cell death. These experimental conditions enhance gene expression for the pro-apoptotic protein Bax and concomitantly decrease expression for Bcl-2 and Bcl-X_L_. Our study indicated for the first time, that the expression of Sphk1, the enzyme involved in pro-survival signalling [45, 46], was significantly increased. However, endogenous synthesis of S1P was not able to protect cells against death. Our study indicated the neuroprotective effect of exogenously applied S1P at a concentration of 1 μM on death signalling in HT22 cell exposed to GD/GR stress. A higher concentration of S1P (10 and 30 μM) induced cell death from about 50 % to 80 %, respectively (data not shown), probably through its degradation to sphingosine and ceramide. S1P, the product of Sphk1/2 activity, is a bioactive lipid mediator that promotes cell survival, proliferation, migration and angiogenesis. Disturbances in gene expression for Sphk1/Sphk2 and sphingosine-1-phosphate receptor 1 (S1P1) were observed in the animal experimental model of cerebral ischemia [47]. Spiegel and Milstien observed that the S1P analogue (PFTY720) reduced neuronal injury, possibly via S1P1 activation [47]. S1P may be also implicated in the neuroprotective effect in acute brain and spinal cord injury with regard to mitochondrial dysfunction and the oxidative stress mechanism [48]. The other in vitro experiments demonstrated that S1P inhibits apoptosis in response to oxidative stress induced by H_2_O_2_ [49]. It was observed that this anti-apoptotic effect of S1P is mediated through PI3K/Akt signalling and is regulated through receptors S1P1 and S1P3. The protective effect of S1P was abolished after treatment with VPC23019, an antagonist of S1P1 and S1P3 receptors, W146, an antagonist of S1P1 receptors, and CAY10444, an antagonist of S1P3 receptors. According to our data the neuroprotective effect of S1P is also mediated by activation of receptors S1P1 and S1P3 in GD/GR toxicity. The S1P analogue P-FTY720 and the specific agonist of S1P1 (SEW2871) enhanced cell viability under GD/GR stress. It was also reported that mitochondria are involved in neuroprotective effect of S1P in oxygen-glucose deprivation model [50]. The data obtained by Agudo-López et al. [50] pointed out that S1P treatment significantly reduces both necrosis and apoptosis in the in vitro model of ischemia. The protection mechanism involved stabilisation of the mitochondrial membrane potential, reduced calcium loading and decreased sensitivity to mPTP opening. Our data suggest that the protective mechanism of S1P is dependent on Bcl-2 proteins, which control a critical step in commitment to apoptosis by regulating permeabilisation of the mitochondrial outer membrane [51]. In our study, S1P regulated pro-survival signalling by enhancing expression of Bcl-2 and Bcl-X_L_; both anti-apoptotic proteins suppressed by GD/GR. Consequently S1P may lead to the stabilization of mitochondrial membrane potential and reduce the ROS production. Anti-apoptotic Bcl-2 family members (Bcl-2, Bcl-X_L_) have a well-described role in scaffolding the Bcl-2 homology (BH3) domain of pro-apoptotic Bcl-2-family members, thereby neutralising their pro-apoptotic activity [52]. According to some other studies, exogenous S1P regulate the expression of pro‑apoptotic and anti‑apoptotic proteins. It has been observed that S1P increases the expression of anti‑apoptotic Bcl-2 [53–55] and Mcl1 [56] as well as down-regulates the pro‑apoptotic proteins Bad and Bax [55, 57–59]. Overexpression of Sphk1 up-regulates Bcl-2 and down-regulates B-cell lymphoma 2 interacting mediator of cell death (Bim) [30, 31]. Similarly, overexpression of Sphk1 inhibits the activation of caspase 3, cytochrome *c* and SMAC/Diablo release from mitochondria through the modulation of Bim, Bcl‑X_L_ and Mcl1 in chronic myeloid leukaemia cells [29]. There are also evidences that Bcl-2 overexpression may stimulate Sphk1 expression and activity in human melanoma cells [60]. Our data indicate that stimulating effect of S1P on the expression of anti-apoptotic Bcl-2 proteins can be an important protective mechanism against GD/GR stress.
